# Outcomes of “sandwich” chemoradiotherapy compared with chemotherapy alone for the adjuvant treatment of FIGO stage III endometrial cancer

**DOI:** 10.3389/fonc.2022.946113

**Published:** 2022-09-23

**Authors:** Shao-Jing Wang, Lily Wang, Lou Sun, Yu-Hsiang Shih, Shih-Tien Hsu, Chin-Ku Liu, Sheau-Feng Hwang, Chien-Hsing Lu

**Affiliations:** ^1^ Department of Gynecology and Obstetrics, Taichung Veterans General Hospital, Taichung, Taiwan; ^2^ Department of Radiation Oncology, Taichung Veterans General Hospital, Taichung, Taiwan; ^3^ Center for General Education, Ling Tung University, Taichung, Taiwan; ^4^ School of Medicine, China Medical University, Taichung, Taiwan; ^5^ Department of Palliative Care Unit, Taichung Veterans General Hospital, Taichung, Taiwan; ^6^ Institute of Biomedical Sciences, Ph.D. Program in Translational Medicine, and Rong-Hsing Research Center for Translational Medicine, National Chung-Hsing University, Taichung, Taiwan

**Keywords:** endometrial neoplasms, FIGO stage III, adjuvant therapy, chemotherapy, radiotherapy, chemoradiotherapy

## Abstract

**Objective:**

To analyze and compare outcomes of adjuvant chemoradiotherapy in patients with International Federation of Gynecology and Obstetrics (FIGO) stage III endometrial cancer (EC) patients using the “Sandwich” sequence and chemotherapy (CT) alone.

**Methods:**

From, 2005 to, 2019, we retrospectively reviewed 80 patients with FIGO stage III EC who received treatment at our institute. We analyzed 66 patients who had undergone complete surgical staging followed by adjuvant treatment with sandwich chemoradiotherapy (39 patients) and CT alone (27 patients). The 5-year overall survival (OS), progression-free survival (PFS), and disease-specific survival (DSS) were calculated using the Kaplan–Meier method. Additional prognostic factors were analyzed using Cox proportional hazards regression.

**Results:**

Herein, the analysis was conducted using 66 patients with a median follow-up period of 50 and 85 months in the sandwich and CT-alone arms. Comparing the sandwich sequence and CT-alone groups, the 5-year OS and PFS were 87% *vs*. 70% (*p* = 0.097) and 77% *vs*. 65% (*p* = 0.209), respectively. The sandwich therapy conferred an improved 5-year DSS (92% *vs*. 70%, *p* = 0.041) and a lower local recurrence rate (0% *vs*. 11%, *p* = 0.031). In multivariable analyses, grade 3 histology and deep myometrial invasion were independent risk factors for 5-year OS and DSS. The sandwich sequence was a positive predictor for 5-year DSS (hazard ratio [HR] = 0.23, *p* = 0.029). The sandwich arm demonstrated higher acute hematologic toxicity than the CT-alone arm. CT dose delay/reduction and treatment completion rates were similar in both groups.

**Conclusion:**

For patients with stage III EC, postoperative sandwich chemoradiotherapy appears to offer a superior 5-year DSS and local control with tolerable toxicity when compared with CT alone.

## Introduction

Endometrial cancer (EC) is the most common gynecologic malignancy with a steadily growing incidence (1). Although most ECs are diagnosed early with a favorable prognosis, approximately 21% of cases are presented as locally advanced diseases (2). A complete staging operation remains the cornerstone of EC management (3). However, the optimal adjuvant therapy for locally advanced ECs is yet to be established.

Following the Gynecologic Oncology Group study (GOG-122), chemotherapy has been established as the mainstay of adjuvant treatment for advanced EC. The study reported a superior PFS and OS when comparing doxorubicin plus cisplatin to whole abdominal radiation (4). However, chemotherapy alone was also associated with a higher local recurrence rate of 20% (5). Recently, two randomized control trials compared different therapies in patients with high-risk EC. The PORTEC-3 trial reported an improved OS and failure-free survival particularly in patients with stage III EC receiving chemoradiotherapy when compared with radiotherapy (RT) alone (6). In the GOG-258 trial, the addition of pelvic irradiation to CT failed to significantly benefit relapse-free survival, while presenting a trend toward improved local control and more distant metastasis (5). Given the increased adverse events following chemoradiotherapy and the lack of evidence supporting its benefit, the role of RT warrants further investigation.

Several studies with large retrospective cohorts from the National Cancer Database (NCDB) have addressed outcomes of different chemoradiotherapy sequences (7–10). As an initial adjuvant modality, potential benefits of systemic CT include early treatment of occult micro-metastatic disease, reduced likelihood of CT delay secondary to RT-related toxicities, and avoiding the potential for RT-induced tumor vascular bed alteration known to impair chemotherapeutic drug delivery to malignant cells (11). Conversely, initial treatment with CT prior to pelvic irradiation may delay local therapy, compromise tolerance to RT toxicity, and potentially induce a negative impact on local recurrence (12).

The sandwich sequence, comprising 2 to 4 cycles of CT followed by irradiation and subsequent CT, has shown promising results in several phase II studies and retrospective cohorts (11–22). However, its efficacy has been inconsistent and was further limited by small study samples, as well as heterogeneous compositions of histology and staging across studies. Herein, our primary objective was to determine the clinical outcomes of sandwich chemoradiotherapy as an optimal adjuvant treatment for locally advanced ECs.

## Materials and methods

### Patient selection

The present study was a single-centered, retrospective review of female patients with stage III ECs treated between, 2005 and, 2019. Following the approval of the institutional review board, we reviewed a tumor registry to identify all patients with pathologically confirmed stage III EC receiving adjuvant therapy at the Taichung Veterans General Hospital. Pathological reports were reviewed and categorized in accordance with the International Federation of Gynecology and Obstetrics (FIGO) 2009 classification.

All enrolled patients had undergone a primary complete staging surgery comprising total hysterectomy (TH; either open or minimally invasive approach), bilateral salpingo-oophorectomy (BSO), bilateral pelvic lymph node dissection (BPLND), with or without para-aortic lymph node dissection (PALND), and omentectomy. Following surgical intervention, adjuvant therapy with either a “sandwich” chemoradiotherapy sequence or CT alone was performed. The exclusion criteria were as follows: patients with gross residual disease >1 cm after primary staging surgery, patients receiving CT or RT prior to surgery, patients with stage III disease established only upon positive peritoneal washings or synchronous ovarian and endometrial cancer, patients treated with palliative intent, and patients concurrently diagnosed with other cancers within 5 years before and after diagnosis of EC. In addition, we excluded patients with a histological diagnosis of carcinosarcoma, undifferentiated and dedifferentiated carcinoma, and any other type of sarcoma.

### Treatment and monitoring protocol

Adjuvant treatments were initiated within 3 weeks postsurgery. All patients were treated according to the consensus of multidisciplinary tumor boards and clinicians’ choice. The sandwich sequence included three consecutive cycles of platinum-based CT at an interval of 21 days, followed by RT and another 3 cycles of CT. In the CT-alone group, patients were treated with platinum-based CT, planned for 6 cycles. One week before initiating each CT cycle, all patients received blood tests including a complete blood count and differential count, along with liver and renal function assessments. Treatment-related toxicity was graded according to the Common Terminology Criteria for Adverse Events (CTCAE v5.0) (23). If a patient experienced grade ≥3 toxicity on blood test assessment, CT was postponed on a week-by-week basis. Delay of treatment was defined as a delay of ≧7 days from the scheduled date of therapy. Patients with treatment-related toxicity that required a delay for ≧4 consecutive weeks were excluded from our analysis.

At the end of adjuvant therapy, patients were followed up with clinical and physical examinations during the first 3 years, which were performed at 3-month intervals and thereafter at 6-to-12-month intervals. Abdominal computed tomography was performed during the first year at 3-to-6-month intervals and thereafter at 12 months. In the event of clinically suspected metastatic diseases, additional imaging was performed, including computed tomography of the chest, abdomen, and pelvis, as well as positron emission tomography. OS was estimated from the time of surgery to the time of death and censored at the date of the last contact. PFS was calculated from the time of surgery to the time of the first recurrence based on imaging evidence, censored at the date of the last outpatient visit. Recurrence at the vagina or pelvis was considered a local recurrence. Patients who had missed a scheduled follow-up were contacted by our gynecologic oncology managers.

Statistical analyses were conducted using the SPSS version 22.0 (IBM Corp., Armonk, NY, USA). Baseline characteristics were compared using the chi-squared or Mann–Whitney U test. OS, PFS, and disease-specific survival (DSS) were estimated using the Kaplan–Meier method, and comparisons between the two treatment groups were performed using the log-rank test. Univariate analyses were used to identify independent risk factors associated with disease outcomes. Variables with a *p*-value <0.10 were first extracted. Subsequently, multivariable analysis was performed using the Cox proportional hazards model to estimate the hazard ratio of each variable and compare outcomes between treatment groups. Treatment-induced toxicity was compared using the chi-squared test.

## Results

### Patient characteristics

Between, 2005 and, 2019, we identified 138 patients diagnosed with FIGO stage III EC. After exclusion, 80 cases were eligible for study inclusion. In total, 10 patients underwent sequential chemoradiotherapy (six consecutive CTs followed by RT, or RT followed by CT) and four received RT alone; these two patient groups were excluded from the study, given their small numbers. Considering the remaining 66 patients, 39 (59.1%) received sandwich chemoradiotherapy and 27 (40.9%) received CT alone.

The most commonly identified histological subtype was endometrioid (43 cases, 65.2%), followed by mix-epithelial (13 cases, 19.7%), serous (8 cases, 12.1%), and clear cell (2 cases, 3.0%). Each enrolled patient underwent BPLND in addition to TH and BSO. Most of these patients (92.4%) also received PALND. The median number of pelvic lymph nodes retrieved was 21 in the sandwich group and 25 in the CT-alone group. Considering para-aortic lymph nodes, 12 and nine nodes were retrieved from the sandwich and CT-alone groups, respectively. **Table 1** summarizes the patients’ baseline characteristics. No difference was detected between the two treatment groups in terms of patient age, surgical stage, histology, and pathological risk factors.

**Table 1:** Characteristics of the patients (N = 66).

	Sandwich (n = 39)		CT alone (n = 27)		*p-*value
Median follow-up interval (months)	50.1	(26.0-77.7)	85.3	(36.9-112.4)	0.035^*^
Age	55.0	(48.0-64.0)	55.0	(47.0-57.0)	0.330
BMI	23.9	(21.3-26.1)	22.9	(18.5-26.2)	0.235
FIGO stage no. (%)^‡^					0.688
IIIA	9	(23.1%)	5	(18.5%)	
IIIB	2	(5.1%)	1	(3.7%)	
IIIC1	15	(38.5%)	8	(29.6%)	
IIIC2	13	(33.3%)	13	(48.1%)	
Histology					0.329
Endometrioid grade 1 and 2	18	(46.2%)	10	(37.0%)	
Endometrioid grade 3	10	(25.6%)	5	(18.5%)	
Serous	4	(10.3%)	4	(14.8%)	
Mixed-epithelial	5	(12.8%)	8	(29.6%)	
Clear cell	2	(5.1%)	0	(0.0%)	
Histology grading					0.609
Grade 1	4	(10.3%)	1	(3.7%)	
Grade 2	14	(35.9%)	10	(37.0%)	
Grade 3	21	(53.8%)	16	(59.3%)	
Gross residual disease					–
Absent	39	(100.0%)	27	(100.0%)	
Present	0	(0.0%)	0	(0.0%)	
No. of dissected lymph nodes					
Pelvic lymph node	21.0	(15.0-32.0)	25.0	(17.0-34.0)	0.270
Para-aortic lymph node	12.0	(6.0-17.0)	9.0	(4.0-14.0)	0.176
No. of cases receiving PALND	36	(92.3%)	25	(92.6%)	1.000
Minimal invasive approach	13	(33.3%)	0	(0.0%)	0.002^†^
LVSI					1.000
Absent	11	(28.2%)	7	(28.0%)	
Present	28	(71.8%)	18	(72.0%)	
Deep myometrial invasion					0.817
Absent	15	(38.5%)	12	(44.4%)	
Present	24	(61.5%)	15	(55.6%)	
Comorbidities					
Hypertension	8	(20.5%)	1	(3.7%)	0.071
Type II DM	8	(20.5%)	4	(14.8%)	0.748
HBV carrier	4	(10.3%)	0	(0.0%)	0.138
Others	6	(15.4%)	5	(18.5%)	0.749
Radiotherapy					–
EBRT dose 46.8 Gy	1	(2.6%)	–		
EBRT dose 50.4–54.0 Gy	33	(86.8%)	–		
EBRT dose >54.0 Gy	4	(10.5%)	–		
Vaginal brachytherapy	13	(33.3%)	0	(0.0%)	0.002^†^
No. of CT cycles					0.460
4–5 cycles	1	(2.6%)	1	(3.7%)	
6 cycle	38	(97.4%)	25	(92.6%)	
>6 cycle	0	(0.0%)	1	(3.7%)	
CT regimen					0.979
Platinum + paclitaxel	29	(74.4%)	21	(77.8%)	
Platinum + doxorubicin	10	(25.6%)	6	(22.2%)	
CT delay or dose reduction					0.795
Absent	23	(59.0%)	17	(65.4%)	
Present	16	(41.0%)	9	(34.6%)	

Chi-square test or Mann–Whitney U test. *p < 0.05, ^†^p < 0.01.

Values are presented as median (interquartile range) or number (%).

^‡^Stages were allocated according to the International Federation of Gynecology and Obstetrics (FIGO) 2009.

CT, chemotherapy; BMI, body mass index; PALND, para-aortic lymph node dissection; LVSI, lymphovascular space invasion; DM, diabetes mellitus; HBV, hepatitis B; ERBT, external beam radiotherapy.

All patients received a platinum-based CT with either carboplatin (area under the curve [AUC] 4–6) or cisplatin (50 mg/m^2^) plus paclitaxel (135–175 mg/m^2^) or epirubicin (60–80 mg/m^2^), or doxorubicin liposome injection (Lipodox^®^) (30 mg/m^2^). The different combinations of chemotherapy regimens were similar in both arms. The median number of chemotherapy cycles per patient was 6 (4–8). In the sandwich sequence, irradiation was initiated within 3 weeks of the third chemotherapy cycle. RT was administered using external beam radiation therapy (EBRT) and delivered with intensity-modulated radiation therapy (IMRT) to the pelvis. The radiation fields were extended to the para-aortic region if metastasis was pathologically confirmed. The majority of these patients received a dose ranging between 5,040 and 5,400 cGy. Patients with cervical stromal invasion received additional vaginal brachytherapy (dose: 400–1,000 cGy. Details of CT and RT are shown in **Table 1**.

### Outcomes

The median follow-up period was 50 months in the sandwich group and 85 months in the CT-alone group (*p* = 0.035). Disease recurrence was documented in 17 patients. These recurrences included 16 cases of distant metastasis, one case with pelvic recurrence, and two cases with concurrent distant and pelvic recurrences. No vaginal recurrence was detected in the present cohort. The most common site of distant metastasis was the lung (five cases, 7.6%), followed by the retroperitoneum (four cases, 6.1%), bone (four cases, 6.1%), and liver (four cases, 6.1%). During the follow-up period, 16 patients died, with 13 attributed to EC.

**Figure 1:**
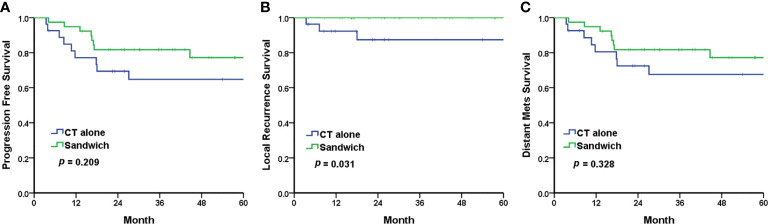
The Kaplan–Meier survival curves for 5-year progression-free survival **(A)**, local recurrence **(B)**, and distant metastasis **(C)**. CT, chemotherapy; mets, metastasis.

**Figure 2:**
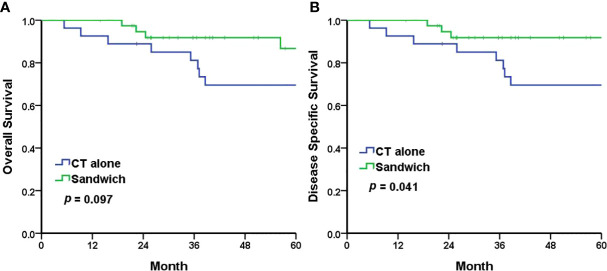
The Kaplan–Meier survival curves for 5-year overall survival **(A)** and 5-year disease-specific survival **(B)**. CT chemotherapy.

The Kaplan–Meier analyses revealed a 5-year PFS of 77.2% and 64.8% in the sandwich and CT-alone groups, respectively (*p* = 0.209) (**Figure 1A**). The sandwich arm was associated with a lower rate of pelvic recurrence than the CT-alone group (0% *vs*. 11.1%, *p* = 0.031) (**Figure 1B**), whereas the PFS for distant metastasis was similar in both groups (77.2% *vs*. 67.6%, *p* = 0.328) (**Figure 1C**). Although the difference in 5-year OS between the two groups did not reach statistical significance (86.7% *vs*. 69.6%, *p* = 0.097) (**Figure 2A**), a significantly improved (DSS) was observed in the sandwich group (91.8% *vs*. 69.6%, *p* = 0.041) (**Figure 2B**).

Based on univariate and multivariable analyses, grade 3 histology and deep myometrial invasion were identified as independent risk factors for 5-year OS and 5-year DSS. The sandwich sequence was a positive predictor for 5-year DSS (HR = 0.23, 0.06–0.86, *p* = 0.029). For PFS, grade 3 histology was the only negative predictor that attained statistical significance (**Table 2**).

**Table 2:** Univariate and multivariable analyses of prognostic factors for 5-year OS, PFS, and DSS.

5-year overall survival															
	Univariate							Multivariable								
	HR		95% CI				*p-*value	HR		95% CI		*p-*value				
Age group																
<60	Reference															
≥60	1.22		(0.33-4.53)				0.763									
FIGO stage																
IIIA and IIIB and IIIC1	Reference															
IIIC2	1.74		(0.56-5.41)				0.337									
Histology grading																
Grades 1 and 2	Reference							Reference								
Grade 3	9.69		(1.25-75.10)				0.030^*^	10.44		(1.34-81.10)		0.025^*^				
Treatment																
CT alone	Reference							Reference								
Sandwich	0.38		(0.11-1.25)				0.110	0.31		(0.09-1.02)		0.054				
LVSI																
Absent	Reference															
Present	4.19		(0.54-32.44)				0.170									
Deep myometrial invasion																
Absent	Reference							Reference								
Present	8.83		(1.14-68.42)				0.037^*^	10.52		(1.35-82.01)		0.025^*^				
BMI																
<25	Reference															
≥25	0.64		(0.17-2.36)				0.502									
Cervical stromal involvement																
Absent	Reference															
Present	1.38		(0.44-4.36)				0.581									
**5-year disease-specific survival**																
		Univariate							Multivariable							
		HR		95% CI		*p-*value			HR		95% CI		*p-*value			
Age group																
<60		Reference														
≥60		1.34		(0.36-5.07)		0.665										
FIGO stage																
IIIA and IIIB and IIIC1		Reference														
IIIC2		1.45		(0.44-4.75)		0.541										
Histology grading																
Grades 1 and 2		Reference							Reference							
Grade 3		8.70		(1.11-68.01)			0.039^*^		9.16		(1.17-71.70)		0.035^*^			
Treatment																
CT alone		Reference							Reference							
Sandwich		0.27		(0.07-1.04)			0.056		0.23		(0.06-0.87)		0.030^*^			
LVSI																
Absent		Reference														
Present		3.82		(0.49-29.89)			0.201									
Deep myometrial invasion																
Absent		Reference							Reference							
Present		7.85		(1.00-61.34)			0.050		9.44		(1.20-74.15)		0.033^*^			
BMI																
<25		Reference														
≥25		0.72		(0.19-2.70)			0.622									
Cervical stromal involvement																
Absent		Reference														
Present		1.11		(0.33-3.81)			0.863									
**5-year progression-free survival**															
Age group															
<60		Reference													
≥60		0.79		(0.23-2.74)			0.705								
FIGO stage															
IIIA and IIIB and IIIC1		Reference													
IIIC2		2.03		(0.78-5.28)			0.145								
Histology grading															
Grades 1 and 2		Reference						Reference							
Grade 3		15.80		(2.09-119.33)			0.007^†^	11.57		(1.52-87.80)		0.018^*^			
Treatment															
CT alone		Reference						Reference							
Sandwich		0.55		(0.21-1.42)			0.216	0.50		(0.19-1.30)		0.155			
LVSI															
Absent		Reference						Reference							
Present		7.31		(0.97-55.16)			0.054	4.54		(0.56-36.87)		0.157			
Deep myometrial invasion															
Absent		Reference						Reference							
Present		1.99		(0.70-5.64)			0.198	1.50		(0.51-4.45)		0.461			
BMI															
<25		Reference													
≥25		1.22		(0.46-3.20)			0.693								
Cervical stromal involvement															
Absent		Reference													
Present		1.23		(0.46-3.33)			0.682								

Cox proportional hazard regression. *p < 0.05, ^†^p < 0.01.

CT, chemotherapy; LVSI, lymphovascular space invasion; BMI, body mass index.

### Treatment-related toxicity

The sandwich sequence was associated with higher incidence and greater severity of neutropenia (grades 3–4: 56.4% *vs*. 18.5%, *p* = 0.005) and hematologic toxicity (grades 3–4: 59.0% *vs*. 25.9%, *p* = 0.016) than the CT-alone group (**Table 3**). Dose reduction was performed in one patient from 50.4 to 46.8 Gy, owing to skin irritation during RT. The proportions of patients requiring a dose delay or reduction during CT (34.6% *vs*. 41.0%, *p* = 0.795) were comparable. Furthermore, treatment completion rates were similar between the two groups (97.4% *vs*. 96.2%, *p* = 0.642). Five patients experienced lymphoceles after receiving RT, and they all resolved spontaneously within 18 months of the follow-up period. No patient reported hematuria during the follow-up. Grade 1–2 hematochezia was documented in five patients, and after medical treatment, no patient experienced sustained hematochezia. No patient died from treatment-associated adverse events in this cohort.

**Table 3:** Adverse events.

	Grades 1–2					Grades 3–4				
Sandwich	(N = 39)	CT alone	(N = 27)	*p*-value	Sandwich	(N = 39)	CT alone	(N = 27)	*p*-value	
Anemia	31	(79.5%)	20	(74.1%)	0.828	3	(7.7%)	2	(7.4%)	1.000
Neutropenia	15	(38.5%)	8	(29.6%)	0.633	22	(56.4%)	5	(18.5%)	0.005^†^
Thrombocytopenia	19	(48.7%)	5	(18.5%)	0.025^*^	3	(7.7%)	0	(0.0%)	0.264
Hematologic toxicity	16	(41.0%)	17	(65.4%)	0.095	23	(59.0%)	7	(25.9%)	0.016^*^
Liver toxicity	15	(38.5%)	3	(11.5%)	0.036^*^	1	(2.6%)	0	(0.0%)	1.000
Renal toxicity	1	(2.6%)	2	(7.7%)	0.559	0	(0.0%)	0	(0.0%)	–

Chi-square test. *p < 0.05, ^†^p < 0.01.

Values are presented as number (%).

CT, chemotherapy.

## Discussion

In the present study, we detected a significant improvement in 5-year DSS and local control in patients treated with the sandwich sequence when compared with those treated with six consecutive cycles of CT alone. Multivariable analyses revealed that sandwich chemoradiotherapy was a positive prognostic factor for 5-year DSS, whereas both grade 3 histology and deep myometrial invasion were negative predictors for 5-year OS and DSS. Moreover, grade 3 histology was associated with a worse 5-year PFS. The proportions of treatment completion were similarly high in both groups, despite a significantly higher incidence and greater severity of neutropenia and hematologic toxicity in the sandwich sequence than in the CT-alone group.

Over the last 3 years, observational cohorts from the NCDB database have examined different sequences of adjuvant treatment. Goodman et al. have reported a longer 5-year OS in patients with stage III–IV, grade I–II endometrioid ECs who were treated with the CT-RT sequence when compared with those treated with RT-CT or either therapy alone (7). In patients with stage IIIC disease, a survival benefit was documented following treatment with the CT-RT sequence when compared with concurrent chemo-radiotherapy (CCRT) (8, 9). Xiang et al. have shown that the addition of pelvic irradiation to CT, irrespective of the sequence, affords a superior survival in patients with stage IIIC2 endometrioid ECs and stage IIIB, IIIC non-endometrioid ECs (10). These results supported the importance of RT as an adjuvant treatment of locally advanced ECs and the trend toward better survival in patients who had upfront CT in their adjuvant treatments.

Sandwich chemoradiotherapy was first reported in two pilot phase II studies. Both studies showed encouraging outcomes for locally advanced EC presenting high-risk histologies (12, 13). Subsequent single-armed studies also revealed a modest efficacy with acceptable toxicity in patient groups exhibiting different stages and histologic compositions (14–17). According to Secord et al., adjuvant sandwich therapy could improve the 3-year OS and PFS when compared with sequential CT-RT or RT-CT in patients with stage III–IV disease (11). In patients with stage III endometrioid EC, Lu et al. have reported comparable OS, PFS, and toxicity between sandwich and sequential chemoradiotherapy. However, the authors found that the group survival outcomes appeared similar, possibly due to small sample sizes (18). In comparison, although the 5-year OS in our study also failed to reach statistical significance between treatment groups, the 5-year DSS was significantly improved in the sandwich arm.

Recently, a multicenter retrospective analysis examining 179 patients with stage IIIC disease reported a significantly improved 5-year OS in the sandwich arm when compared with the sequential arm (74% *vs*. 56%). A trend toward a better PFS (65% *vs*. 54%, *p* = 0.05) was also reported (19). In a later cohort study assessing the same group of patients, the authors also identified a better 5-year OS (62% *vs*. 35%) and PFS (57% *vs*. 35%) using subgroup analyses among stage IIIC2 patients treated with a sandwich sequence when compared with sequential chemoradiotherapy (20). In addition, McEachron et al. have demonstrated OS and PFS benefits in patients with stage III–IV EC treated with sandwich therapy when compared with those treated with alternate sequences (21). In this multicenter analysis assessing 152 patients with relatively poor histology, 44% had endometrioid, 47.5% presented serous EC, and 8.5% had clear cell EC. With 20% of patients exhibiting stage IV disease, the authors found a 3-year OS advantage in the sandwich group when compared with CT-RT and RT-CT (71% *vs*. 52% *vs*. 50%), along with similar results for 3-year PFS (55% *vs*. 34% *vs*. 37%). In a more recent cohort study by Ko et al., using the SEER-Medicare database, the authors identified 44 cases treated with sandwich therapy in a subclassification analysis out of 2,870 patients with stage III disease (22). The best 5-year OS was observed in endometrioid EC treated with the sandwich regimen (82%), serous EC treated with the CCRT regimen (48%), and clear cell EC treated with the CCRT regimen (66%). These comparative cohorts demonstrated promising results corroborating the efficacy of the sandwich sequence. However, prospective randomized control trials are warranted to further validate its efficacy.

On the other hand, GOG-258 failed to display a superior relapse-free survival with chemoradiotherapy when compared with CT alone (59% *vs*. 58%, *p =* 0.20) in patients with stage III–IVA EC. Although the chemoradiotherapy group was associated with improved local control exhibiting fewer pelvic/para-aortic (11% *vs*. 20%) and vaginal recurrences (2% *vs*. 7%), more distant recurrences were also detected (27% *vs*. 21%). The chemoradiotherapy protocol consisted of RT, with 2 cycles of concurrent cisplatin, followed by 4 cycles of CT with paclitaxel plus carboplatin. Data on this combination of CCRT plus CT remain limited. In the study by Ko et al. assessing 2,870 patients with stage III EC from the SEER-Medicare database, the authors identified <11 patients receiving CCRT plus CT, similar to that reported in GOG-258 and PORTEC-3 trials (22). Although CCRT alone was found to afford improved local control (24), recent large retrospective cohorts have reported less favorable survival when compared with the CT-RT sequence (8, 9). In the GOG-258 trial, the higher incidence of distant metastasis observed in the chemoradiotherapy group could be associated with the two fewer cycles of carboplatin and paclitaxel administered, as the two cycles of cisplatin cannot be regarded as equally potent to carboplatin plus paclitaxel. Moreover, the chemoradiotherapy arm was found to exhibit a lower CT completion rate (75% *vs*. 85%). Given that these results from recent large cohorts indicate the importance of upfront CT, the chemoradiotherapy sequence administered in GOG-258 appeared to be a relatively suboptimal choice. In contrast, several comparative cohorts have reported a superior survival benefit with the sandwich regimen over sequential chemoradiotherapy. Whether the sandwich sequence affords additional survival benefits in patients with stage III EC when compared with CT alone warrants further investigation.

In the current study, we excluded patients with gross residual tumors to ensure that both arms were comparable in postsurgical status before initiating adjuvant therapy. Extensive lymph node dissection reportedly affords a survival benefit in locally advanced endometrioid EC (25–27). Algkiozidis et al. have reported improved survival in patients undergoing dissections of ≧17 lymph nodes (26). In the present cohort, all patients had received BPLND, with PALND performed in >90% of patients. The median number of lymph nodes removed was 33 and 34 in the sandwich sequence and CT-alone groups, respectively. Given the extent of lymph node dissection, we aimed to achieve a complete excision of all metastatic lymph nodes. Hence, the risk of missing occult metastasis was minimized, facilitating the determination of precise areas for adjuvant RT, thus more accurately reflecting its efficacy.

After, 2012, minimally invasive approaches for preoperatively suspected early-staged EC were widely employed at our institution. This explains why the sandwich group comprised patients staged with the minimally invasive approach. No difference was detected in terms of OS, DSS, and PFS between the different surgical approaches. Furthermore, our preference for adjuvant therapy had shifted since, 2010 as growing numbers of publications have supported the efficacy of sandwich chemoradiotherapy. All patients in the sandwich arm were treated after, 2010; in the CT-alone arm, 17 patients (63%) were treated after, 2010. CT regimens in our patients were either carboplatin plus paclitaxel or carboplatin/cisplatin plus epirubicin/Lipodox^®^, with equal combinations in both groups. The choice of chemotherapeutic regimen has remained unaltered over the 15-year span. Given that these combinations provide a similar potency and treatment completion rate (28), the non-uniformity of CT regimens should minimally impact our results.

Hematologic adverse events were the most common cause of a CT dose delay or reduction in the current study. Previous cohorts have reported a dispersed level of toxicity with the sandwich therapy. However, most of these studies failed to specify their surveillance protocol during treatment. Onal et al. have documented a considerably low toxicity profile, with >grade 2 neutropenia observed only in 9% of cases treated with sandwich sequence (19). Frimer et al. have reported a 35% incidence of >grade 2 hematologic toxicity, as estimated by CT cycles rather than the proportion of patients (16). In our analysis, the rates of grade 3–4 hematologic toxicity were 59% in the sandwich arm and 26% in the CT-alone arm; in GOG-258, these rates were 40% for CCRT plus CT and 52% for CT alone. Despite the significant bone marrow toxicity noted in the sandwich arm of our study, both groups exhibited a comparable rate of CT dose delay or reduction (41.0% *vs*. 34.6%, *p* = 0.795), as well as treatment completion rate (97.4% *vs*. 96.2%, *p* = 0.642). Accordingly, although incorporating irradiation does increase toxicity, the adverse events were eventually tolerable in most of our patients.

The major limitation of the present study is its retrospective nature and limited sample size collected from a single institution. Selection bias is also a concern, as patients exhibiting high risks or superior performance status are likely to receive more aggressive adjuvant treatments. Nevertheless, we analyzed potential risk factors and did not identify any selection bias. Secondly, our cases were reviewed over a span of 15 years, during which the routine practice and clinician preferences were likely altered. Our institute initiated adjuvant sandwich chemoradiotherapy only after, 2012, resulting in imbalanced monitoring times between the two treatment groups. The more recently enrolled patients likely benefited more from newly developed treatment modalities. Thus, these treatment modalities may have lengthened patient life spans after recurrence. In addition, while most retrospective studies addressing sandwich chemoradiotherapy have examined OS and PFS as their primary outcome, our study did not detect a significantly improved 5-year OS in the sandwich group, although the 5-year DSS showed improvement. Furthermore, outcomes of our cohort were likely improved owing to the exclusion of carcinosarcoma and undifferentiated and dedifferentiated carcinoma in both arms. The latter two histologies, which are less specifically described and excluded in other studies, also carry a distinctly poor prognosis. Finally, the toxicity profile can only be assessed by reviewing the laboratory tests and charts during adjuvant treatment. Chronic toxicity could not be reliably assessed as documentation of symptoms may be inconsistent among clinicians, and the reporting bias of patients may also affect outcomes. Therefore, the current study did not analyze neurotoxicity, constitutional symptoms, and other late events. The strength of our study is the uniformity of postsurgical status and the extent of lymph node assessment. Over 90% of our patients underwent PALND, which possibly reduced occult para-aortic metastasis and more appropriately reflected the efficacy of RT. Despite a significantly shorter follow-up period in the sandwich arm, both arms were monitored for a longer period when compared with other cohort studies. Furthermore, compared with the 75% and 85% chemotherapy cycle completion rates reported in GOG-258, almost all our patients completed their scheduled treatment. Hence, our results may better reflect the true potency of both treatment arms.

In conclusion, we documented a better 5-year DSS and local control in the sandwich chemoradiotherapy sequence than in the CT-alone group. The sandwich sequence was associated with increased hematologic toxicity, which appeared tolerable in most patients and did not impact the treatment completion rate. To the best of our knowledge, this is the first study that directly compared the sandwich sequence with CT alone. As survival outcomes are yet to be established in the GOG-258 trial, the survival benefits shown in our study provide additional information supporting the efficacy of sandwich chemoradiotherapy. Further prospective randomized studies are required to validate the efficacy of the sandwich regimen and identify the optimal adjuvant therapy.

## Data availability statement

The original contributions presented in the study are included in the article/**Supplementary Material**. Further inquiries can be directed to the corresponding author.

## Ethics statement

This study was reviewed and approved by Institutional Review Board I &II of Taichung Veterans General Hospital. Written informed consent for participation was not required for this study in accordance with the national legislation and the institutional requirements.

## Author contributions

The authors confirm their contribution to the paper as follows: S-JW and C-HL were responsible for the study conception, design, and draft manuscript. LW, LS, Y-HS, C-KL and S-FH were responsible for data acquisition. S-TH and C-HL helped with data interpretation and performed statistical analyses. S-JW, LS and Y-HS wrote the original draft of the manuscript. All authors contributed to the article and approved the submitted version.

## Funding

This work was supported by Taichung Veterans General Hospital and National Chi Nan University. (Grant number: TCVGH-NCNU1117903).

## Acknowledgments

The authors thank the Biostatistics Task Force of Taichung Veterans General Hospital and the Taiwan Society of Cancer Registry for their assistance.

## Conflict of interest

The authors declare that the research was conducted in the absence of any commercial or financial relationships that could be construed as a potential conflict of interest.

## Publisher’s note

All claims expressed in this article are solely those of the authors and do not necessarily represent those of their affiliated organizations, or those of the publisher, the editors and the reviewers. Any product that may be evaluated in this article, or claim that may be made by its manufacturer, is not guaranteed or endorsed by the publisher.
