# Supplementary material for: Outcomes of “sandwich” chemoradiotherapy compared with chemotherapy alone for the adjuvant treatment of FIGO stage III endometrial cancer
Source: Front Oncol. 2022 Sep 23;12:946113. doi: 10.3389/fonc.2022.946113 (PMC9538654; doi:10.3389/fonc.2022.946113)
Supplement: Supplementary file 1 [file DataSheet_1.pdf]

Supplementary materials

	Before 2010	2010 and beyond
Sandwich		
Platinum + Paclitaxel	0	30
Platinum + Anthracycline	0	9
CT alone		
Platinum + Paclitaxel	7	14
Platinum + Anthracycline	3	3
